# Orbital Mucosa-Associated Lymphoid Tissue Lymphoma and Primary Cutaneous Classical Hodgkin Lymphoma: A Rare Case Report and Review of the Literature

**DOI:** 10.1155/2020/1945058

**Published:** 2020-03-28

**Authors:** Nicole Yun, James Coggan, Ira Miller, Parameswaran Venugopal

**Affiliations:** ^1^Department of Internal Medicine, Rush University Medical Center, 1717 W. Congress Parkway, 1042 Kellogg, Chicago, IL 60612, USA; ^2^Division of Hematology, Oncology and Cell Therapy, Rush University Medical Center, Chicago, IL, USA; ^3^Department of Pathology, Rush University Medical Center, Chicago, IL, USA

## Abstract

A 60-year-old woman was diagnosed with isolated mucosa-associated lymphoid tissue (MALT) lymphoma of the ocular adnexa and treated with two years of weekly rituximab for eight doses followed by rituximab maintenance. After nearly two years of maintenance therapy, she developed a tender, indurated mass on the left neck. Biopsy results were consistent with primary cutaneous classical Hodgkin lymphoma (PCCHL).

## 1. Introduction

Classical Hodgkin lymphoma (HL) with cutaneous involvement is well described in the literature and usually occurs late in the course of the disease. The incidence has decreased in the recent decades, likely owing to improved chemotherapy and radiation therapy of patients with Hodgkin lymphoma, as well as the advent of stem cell transplantation [1]. PCCHL is extremely rare, with only 13 previous cases reported in the literature to date [2, 3]. It occurs when cutaneous Hodgkin lymphoma (CHL) arises in the skin as the primary tumor site, without evidence of systemic disease or of regional nodal involvement.

Non-Hodgkin lymphoma (NHL) and HL can present in the same host, either synchronously or metachronously and perhaps even in composite specimens. We describe the unique case of a patient initially treated for orbital MALT lymphoma who later presented with PCCHL. The combination of MALT lymphoma in the ocular adnexa followed by the appearance of PCCHL without any nodal or bone marrow involvement is exceedingly rare. To our knowledge, this is the first reported case of these two distinct entities occurring in the same host.

## 2. Case History

A 60-year-old woman presented with a 3-week history of left upper eyelid bruising and swelling without constitutional symptoms. Magnetic Resonance Imaging (MRI) revealed a 2.0 cm × 1.3 cm soft tissue mass in the medial extraconal compartment involving the oblique muscle. Biopsy was consistent with extranodal MALT-type marginal zone B-cell lymphoma. Staging workup with computed tomography (CT) and positron-emission tomography (PET) suggested possible cervical and inguinal lymph node involvement. Bone marrow biopsy did not show definitive evidence of involvement. The patient underwent treatment with weekly rituximab monotherapy for 8 weeks. Restaging PET of the orbital soft tissue mass demonstrated decrease in SUV from 11.2 to 4.7 in the orbital soft tissue mass and resolution of the hypermetabolic lymph nodes, indicating clinical and metabolic response to therapy. She was continued on maintenance rituximab every 2 months for 2 years.

**Figure 1:**
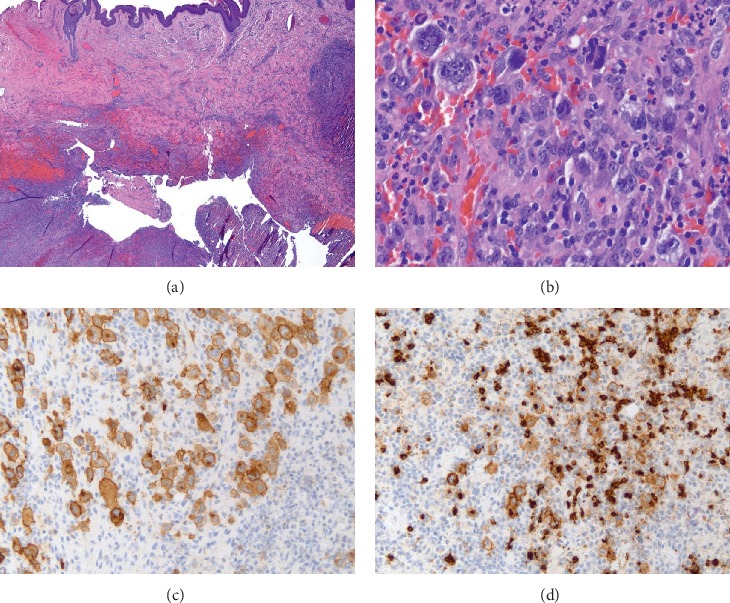
The specimen contained a cystic mass in the dermis, with overlying normal epidermis (a). The cystic areas were surrounded by neutrophils, histiocytes, and large binucleated and multinucleated malignant cells with Hodgkin and Reed–Sternberg morphology (b) that were positive for CD30 (c) and CD15 (d) on immunostains. CD15 also stains admixed neutrophils.

The patient presented to clinic for her last dose of maintenance rituximab with a 3-4 cm tender, indurated, and slightly erythematous mass on the left side of her neck. She denied constitutional symptoms. CT of the neck demonstrated a soft tissue mass on the left lateral aspect of her mid-neck extending into the overlying subcutaneous tissue and skin that was not seen on prior imaging. Excisional biopsy revealed tumor composed of large pleomorphic malignant cells and admixed inflammatory cells. The tumor was centered in the deep dermis, with satellite nodules (mainly microabscesses) in the superficial dermis. No evident lymph nodes were seen in the specimen. Immunohistochemical (IHC) staining showed that the malignant cells were strongly positive for CD30, CD15, PAX5, and CD79a and negative for CD45, diagnostic of classical Hodgkin lymphoma (Figure 1). Oct2 stained a minority of the malignant cells and BOB1 was completely negative, further supporting the diagnosis. Lack of CD20 and EBER excluded morphological variants of diffuse large B-cell lymphoma (DLBCL) and Epstein–Barr virus- (EBV-) positive mucocutaneous ulcer, and the absence of overlying skin ulceration also ruled out the latter. Interesting, the tumor cells expressed CD79a and partial CD2 which are unusual but not impossible findings in this entity. Staging PET-CT and bone marrow biopsy were negative for nodal or systemic involvement. She was treated with 2 cycles of ABVD followed by radiotherapy with no evidence of disease seen on restaging PET.

## 3. Discussion

Patients who have NHL have an increased incidence of developing HL years later. NHL has most commonly been associated with DLBCL, followed by follicular lymphoma. Proposed mechanisms include long-term effects of lymphoma treatment, immune defects, genetic susceptibilities, lifestyle or environmental factors, and likely interactions between these factors [4–6]. There is suspicion of a clonal link between HL and NHL with studies evaluating rare patients with both HL and DLCBL demonstrating that the Hodgkin/Reed–Sternberg cells and the DLBCL cells contain identical VDJ rearrangements of the Ig heavy chain (IGH) locus and contain shared somatic mutations within the VDJ region [7]. In 2016, Alvarez et al. described a woman who presented sequentially with splenic marginal zone lymphoma, Hodgkin lymphoma, and then DLBCL (also thought to be derived from MZL) [8]. Subsequent analysis of clonally rearranged IGH genes in the study revealed that the three histological types of lymphoma had the exact same clonal IGH rearrangement. We did not have enough evidence in our based-on-immunohistochemical staining to suggest that the PCCHL had involvement with prior diagnosis of MLZ/MALT-L. The specimens were never sent out for further molecular testing, and thus a definitive clonal relationship between the two processes could not be established.

13 cases of PCCHL have been reported to date (summarized in Table 1) with treatment and outcomes, if known. The cases have been described as idiopathic as well as iatrogenic. There are no clear guidelines on management and how much treatment is necessary. Previous cases of idiopathic PCCHL have been treated with topical corticosteroids, systemic combination chemotherapy, and/or radiotherapy with varied responses. Iatrogenic PCCHL has been linked to EBV infection, and treatment consisted of withholding immunosuppression.

Our patient was treated with rituximab induction and nearly completed maintenance therapy at the time she was diagnosed with PCCHL. We considered the possibility of iatrogenic PCCHL, as rituximab induces a transient B-cell depletion and dose-dependent T-cell inactivation that could impair T-cell immunosurveillance. This could provoke development and progression of certain second primary malignancies (SPMs), including hematologic malignancies. However, there is no evidence that rituximab specifically contributes to later development of HL [15, 16], especially when EBV has been ruled out as a mediator. Furthermore, our patient was immunocompetent at the time of second lymphoma diagnosis. Complete blood count with differential showed WBC 7.74 K/*μ*L, hemoglobin 13.4 g/dL, platelet count 185 × 10^9^/L, absolute neutrophil count 5570/*μ*L, and absolute lymphocyte count 1110/*μ*L. Serum protein electrophoresis was not obtained. There were no clinically significant cytopenias and no infectious complications throughout the entire course of rituximab treatment and maintenance.

The only reported case of MALT with an extranodal HL was described in 2010. Oka et al. reported a patient with synchronous gastric MALT diagnosed at the same time as primary pulmonary Hodgkin lymphoma. Of note, this patient was infected with EBV, a known predisposing risk factor for Hodgkin lymphoma [17]. Both HRS-like and lacunar cells in the lung tumor and centrocyte-like cells in the MALT lymphoma expressed EBER, so it is likely that EBV was involved in the pathogenesis of both. EBV was excluded in our case.

In accordance with the Lugano Classification System, our patient is considered to have stage IE HL. Treatment rationale was based on National Comprehensive Cancer Network (NCCN) guidelines, which suggests treating early stage HL with extranodal involvement with two cycles of ABVD followed by either two additional cycles or radiation therapy. She tolerated and achieved complete remission with this treatment modality. We will continue to follow her progress.

## 4. Conclusion

In conclusion, PCCHL is an extremely unusual form of extranodal HL. As more reports begin to surface over the centuries, it becomes important to recognize potential risk factors that may be contributing to its pathogenesis, such as immunomodulating therapy, history of NHL, and viral infection/reactivation. Close longitudinal follow-up is recommended in order to identify systemic recurrence of disease, even after achieving remission. This will also lead to better understanding of treatment outcomes, prognostic factors in PCCHL, and primary extranodal HL as a whole and hopefully allow a more uniform consensus regarding therapy.

## Figures and Tables

**Table 1:** Current reported cases of primary cutaneous classical Hodgkin lymphoma, summarized from Koch et al. [3] and Kerstetter [2] with additions.

Reference	Age/sex (M)	PMHx requiring IS	Disease site	Therapy	Outcome	EBV
Idiopathic						
[9]	50	None	Chest wall	MOPP-ABVD	Systemic disease after 2 months	N/A
	54	None	Left lower leg	RT	Systemic progression after 6 years	N/A
	45	None	Forearms and legs	Multiple chemotherapy	No progression at 5 years postchemo	N/A
	17	None	Right thigh	Topical CT	No progression at 9 years	N/A
	52	None	Right arm	None	No progression at 9 years	N/A

[10]	86	None	Left ankle	RT	Systemic disease discovered after 1 year	N/A
[11]	59	None	Left flank, inner thigh, and right foot	ABVD	No progression at 3 years	N/A
[12]	70	None	Right back	RT	No progression at 7 years	Negative
[3]	49	None	Right forearm	ABVD	Response to treatment but passed away from pulmonary tuberculosis	Negative
[13]	80	None	Left thigh	ABVD	No long-term follow-up data	N/A

Iatrogenic						
[14]	74	UC on infliximab	Scalp	Withholding immunosuppression, excision and chemo	Disease free at 10 months	Positive
[10]	25	DM, on MTX/thalidomide/steroids	Scalp	Withholding immunosuppression, chemo & radiation	Complete remission at 6 years	Positive
[2]	58	DM, on MTX/CellCept/steroids	Elbow	Withholding immunosuppression, excision	Recurrence after 13 months	Positive

IS, immunosuppression; MOPP, mechlorethamine, vincristine, procarbazine, prednisone; ABVD, adriamycin, bleomycin, vinblastine, dacarbazine; RT, radiation therapy; CT, corticosteroids; UC, ulcerative colitis; DM, dermatomyositis; MTX, methotrexate; N/A, status not available.
